# Limb salvage in traumatic hemipelvectomy: case series with surgical management and review of the literature

**DOI:** 10.1007/s00402-023-04913-y

**Published:** 2023-06-14

**Authors:** J. Herold, D. Notov, C. Reeps, K. D. Schaser, K. Kamin, M. Mäder, C. Kleber

**Affiliations:** 1 University Center of Orthopaedic, Trauma and Plastic Surgery, University Hospital Carl Gustav Carus, Dresden, Germany; 2 Department of Visceral, Thoracic and Vascular Surgery, University Hospital Carl Gustav Carus, Dresden, Germany; 3 Department of Orthopedic, Trauma and Plastic Surgery, University Hospital of Leipzig, Liebigstr. 20, 04103 Leipzig, Germany

**Keywords:** Traumatic hemipelvectomies, Limb salvage, REBOA, Pelvic injuries, Quality of life, Long-term outcome

## Abstract

**Background:**

Traumatic hemipelvectomies are rare and serious injuries. The surgical management was described in several case studies, with primary amputation often performed to save the patient's life.

**Methods:**

We report of two survivors with complete traumatic hemipelvectomy resulting in ischemia and paralyzed lower extremity. Due to modern emergency medicine and reconstructive surgery, limb salvage could be attained. Long-term outcome with quality of life was assessed one year after the initial accident.

**Results and conclusions:**

The patients were able to mobilize themselves and live an independent life. The extremities remained without function and sensation. Urinary continence and sexual function were present and the colostomy could be relocated in both patients. Both patients support limb salvage, even having difficulties and follow-up treatments. Concomitant cases are required to consolidate the findings.

**Level of evidence:**

IV.

## Introduction

Complete disruption of the hemipelvis from the axial skeleton through the symphysis pubis and sacroiliac joint with damage to the neurovascular bundle, resulting in paralysis and ischemia of the lower extremity, are referred to as complete traumatic hemipelvectomies [1–4]. These injuries are often associated with open or closed injuries to the peripelvic soft tissues, gastrointestinal tract, and urinary tract [1–3]. They represent a rare but severe injury with a poor prognosis. According to recent literature, limb salvage cannot be achieved in most cases (> 94%) [4].

Traumatic hemipelvectomy (THP) was first described by McPherson in 1960, a case of a patient involved in a motor vehicle accident [5]. Since then, other case reports have been published and the mechanisms of trauma were analyzed and described by Beal and Blaisdell et al. in 1989. They assumed three main mechanisms of trauma: direct avulsion of the lower extremity, extreme external rotation and abduction forces, or massive crush trauma [6].

The incidence of traumatic hemipelvectomies is reported to be 0.6–2.4% of all pelvic trauma [7–9]. To date, most surgical treatment strategies for these injuries have been based on case reports and small studies [9].

He et al. performed the most comprehensive study to date and pointed out that established therapeutic protocols are still lacking because it is not possible to perform prospective or comparative studies. The authors created a flowchart of the treatment process for partial traumatic hemipelvectomy [7].

The first distinction between partial and complete traumatic hemipelvectomy was proposed by Klingman et al. and referred to the extent of injury to the iliac vessels [4]. He et al. defined partial traumatic hemipelvectomy in their study based on a complete tear of the hemipelvis of the SI-joint, discontinuity of the main blood flow due to tearing or thromboembolic blockage, avulsion or severe distension of the lumbosacral plexus, and a lower limb more than 50% connected to the trunk by soft tissues. Although the study population included 21 cases, a high number regarding this rare injury, only in 4 patients the limb could successfully be preserved [7].

On the contrary, some authors advocate early secondary amputation on the assumption that limb-salvage procedures may endanger the patient’s life [9]. Especially in traumatic hemipelvectomy with extensive soft tissue injury and traumatic coagulopathy, bleeding may be difficult to control. In such cases, completion of the hemipelvectomy is a life-saving intervention [4, 6, 10–13].

Taking into consideration that most recommendations regarding the treatment of traumatic hemipelvectomy are based on case reports and small studies over a 60-year period [1–7, 9–23], it is of critical importance to reevaluate these findings regularly. The treatment of polytraumatized patients advanced tremendously during the last decades, particularly the concept of damage control, mass transfusion protocols, and organ replacement therapies.

Nowadays, in complex pelvic injuries with no compressible truncal hemorrhage, open surgery or interventional radiology is usually performed to control the bleeding and prevent exsanguination. Especially in cases with closed peripelvic soft tissue injuries, resuscitative endovascular balloon occlusion of the aorta (REBOA) is increasingly used as a “noninvasive clamp” of the infrarenal aorta (zone 3) to stabilize the patient's hemodynamics in the emergency department until definitive hemostasis can be achieved [24–27].

In the present case series, we aim to summarize the different treatment options described in the literature and present 3 patients (2 complete/1 partial) with traumatic hemipelvectomy (Tables 1 and 2).

**Table 1:** Overview of the sustained injuries of the patients which were diagnosed in the emergency department

Sustained injuries	Patient 1	Patient 2	Patient 3
Head and spine	• Subdural hematoma right • Non-displaced fractures of the proc. transversus L 2–4, left	• Non-displaced fractures of the proc. transversus L4, both sides, L5 right	• None
Thorax	• Pneumothorax, left • Fracture of the 1st rib, right	• None	• None
Abdomen	• Tear of the mesocolon, caecum and colon ascendens • Laceration of the spleen	• Liver contusion • Spleen contusion	• Tear of the colon sigmoideum • Spleen contusion
Pelvis	• Pelvic fracture 61-C3.2 c, h, j, left	• Pelvic fracture 61-C3.2 a, d, j, left • Acetabulum fracture 62-A1.2, right	• Pelvic fracture 61-C3.1 b, d, h, j, left
Uro-genital tract	• Tear of the urethra, bladder and vagina	• Tear of the urethra and bladder • Complete rupture of the pelvic floor	• None
Soft tissues	• IIIC open fracture, left pelvis • C3 decollement injury over the lumbar spine	• IIIC open fracture, left pelvis • Morel-Lavallée injury of the left pelvis and left thigh • C2 soft tissue damage, left tibia	• C3 soft tissue damage, left pelvis
Extremities	• None	• Tibia fracture 42-B3, left • Posterior hip dislocation, right	• None
Vessels	• Tear of the internal iliac and medial gluteal artery as well as dissection of the common iliac artery, right • Tear of the external iliac, femoral and obturator vein, left	• Tear of the external iliac artery and vein, left	• Dissection of the external iliac and tear of the internal iliac artery, left • Tear of the external iliac and femoral vein, left
Nerves	• Tear of the femoral nerve, left	• Tear of the lumbosacral plexus and femoral nerve, left	• Tear of the sciatic and femoral nerve, left

Fracture classification of pelvic and acetabular injuries acc. to AO, open and closed soft tissue damage acc. to Gustilo and Anderson and Tscherne and Oestern, respectively [28, 29]

**Table 2:** Presentation of the relevant scores to classify the injury severity (ISS—Injury Severity Score, NISS—New Injury Severity Score), the probability of a mass transfusion due to massive hemorrhage (TASH—Trauma-associated severe hemorrhage score) [30–32]

Score	Patient 1	Patient 2	Patient 3
ISS	50	43	45
NISS	57	25	57
TASH	22	24	21

## Methods and patients

Several authors have used different definitions to describe partial and complete traumatic hemipelvectomy [4, 19, 21, 22]. The present case series followed the approach of Klingman et al. and defined traumatic hemipelvectomy as follows [4]:

1. Complete disruption of the hemipelvis from the axial skeleton through the symphysis pubis or pubic rim and posterior pelvic ring through a sacroiliac joint luxation or a trans-sacral or trans-iliac fracture
2. Severe injury to the iliac vessels resulting in discontinuity of the main blood flow
  - **Partial**: avulsion, dissection, or disruption of the iliac vessels without lower extremity ischemia
  - **Complete**: including the external iliac artery, resulting in ischemia of the lower extremity
3. Disruption or avulsion of the concomitant lumbosacral plexus or femoral nerve

The distinction between complete THP and partial THP was defined on the criteria of the accompanied vascular injury with ischemia of the lower leg.

General patient demographics as well as prehospital emergency care, time management, bleeding control, early osteosynthesis strategies, transfusion analysis, time management, intensive care unit stay, revision surgery, complications, and outcome were evaluated and are also shown in Table 3.

**Table 3:** General perioperative patient characteristics, incl. classification of hemipelvectomy types, macrohemodynamic data, and initial blood chemistry profiles, administered tranexamic acid

	Patient 1	Patient 2	Patient 3
Trauma mechanism	Pedestrian hit by a truck	Bicyclist hit by a truck	Crushed by a tree
Type of traumatic hemipelvectomy	Partial	Complete	Complete
Age	45	25	46
Gender	F	F	M
Rescue time in min	49	43	97
Technical rescue	–	+	+
Pelvic binder	+	–	+
Prehospital CPR	+	+	–
Prehospital intubation	+	+	–
RR < 90 mmHg	+	+	–
Tranexamic acid	–	–	1 g
Initial lactate in mmol/l	15	9	12
Initial Hb in mmol/l	5	4,7	6,9
Affected side	Left	Both	Left
Bleeding control	Clamping/packing/coiling	Clamping	Packing/REBOA
Initial fixation	c-clamp/external fixator	External fixator/K-wires/plate osteosynthesis	Plate osteosynthesis
Definitive fixation	–	SI-screw, plate osteosynthesis acetabulum	SI-screw, plate osteosynthesis ileum
Revisions/second looks	5	16	9
ICU stay in days	4	56	43
Blood transfusions	96	28	111
Plasma transfusions	99	20	59
Complications	Non controllable, non-occlusive mesenteric ischemia	Wound infection	Compartment syndrome, wound infection, necrosis
Outcome	Death	Limb salvage	Limb salvage

*CPR* cardio-pulmonary resuscitation, *RR* “Riva-Rocci” blood pressure, *ICU* intensive care unit, *c-clamp* compression clamp, *k-wires* Kirschner-Wires, *SI-screw* Sacroiliac-Screw, *REBOA* resuscitative endovascular balloon occlusion of the aorta

The two long-term surviving patients (2, 3) were examined and interviewed one year after the accident. Quality of life was assessed using the Glasgow Outcome Scale (GOS), POLO-, EuroQol TOP-module and the SF-36 chart [33]. Psychological risk of post-traumatic stress disorder was additionally measured using the IES-R score [34]. Lower extremity function was assessed using standardized forms. In addition, radiographs and photographs of the lower extremity were obtained.

The patients have been informed that their cases would be submitted for publication, and they provided their written consent. Statistical analysis was performed using Microsoft Excel^®^ (version 16.35, Microsoft^®^, Redmond, Washington, USA) and GraphPad Prism^®^ (version 8.0; San Diego, CA; USA). The means (MV), standard deviations (SD), medians, and interquartile range (IQR 25–75%) and 95% confidence interval of the means were calculated.

## Results

In the present case series, we retrospectively reviewed the medical records of three patients who met the diagnostic criteria of partial and complete traumatic hemipelvectomy at our level 1 trauma center between 2016 and 2020.

### Pre-hospital emergency treatment

All of the above-mentioned patients were assessed and treated at the scene of injury by a trained emergency physician and paramedics according to the prehospital Advanced Trauma Life Support guidelines [35]. The airway was secured via intubation in patients 1 and 2. One patient had prehospital traumatic cardiac arrest, which was treated according to the European Resuscitation Council guidelines [36]. All patients showed clinical signs of hemorrhagic shock, pale, cold, and mottled skin, and systolic blood pressure below 90 mmHg. Volume treatment was initiated after at least two i.v. lines had been placed and tranexamic acid had been administered in patient 3 (see Table 3). Following the concept of permissive hypotension, care was taken not to significantly exceed the systolic blood pressure above 90 mmHg.

Pre-hospital external stabilization of the pelvis was achieved in two of three patients by the application of a pelvic binder. In patient 2, the soft tissues were severely damaged, and the inguinal vessels were already visible on the scene. Manual groin compression by the paramedics was applied.

Although 2 patients were entrapped (2, 3) and a technical rescue by the fire department was required, the time on scene remained less than one hour. The transport time, i.e., the time from arrival of rescue teams at the accident scene to arrival at the hospital, was (median 34 min; range 31–76 min) and exceeded 1 h in patient 3. This patient required complex technical rescue measures with heavy equipment and long transport due to the remote site of the accident.

### Acute management and early surgical strategies

Mass transfusion protocol was triggered and administered with blood and plasma transfusions in a 1:1 ratio, supplemented by the administration of 4 g fibrinogen and 1 g tranexamic acid in all patients. Patients were examined clinically, checked for additional injuries, e-FAST was used to detect free fluid, radiographs and a multi-slice image CT scan with contrast were performed.

Patient 1 was admitted initially in hemodynamic shock and immediately suffered traumatic cardiac arrest. Resuscitative measures were performed in the emergency department and return of spontaneous circulation (ROSC) was achieved after two minutes, followed by the onset of venous hemorrhage from the exposed iliac vessels in the groin on the left side. The polytrauma-CT performed immediately afterwards confirmed the suspected diagnosis of concomitant injury to the vascular axis, as a non-occlusive dissection of the common iliac artery was found on the right side. Reduction followed by the application of an external pelvic fixator and a pelvic c-clamp were performed for fracture compression.

As hemodynamic instability persisted, additional retroperitoneal packing and open clamping of the external iliac vein and the common femoral vein were required. After the patient stabilized, she was transferred to the hybrid operating theater for angioembolization. Coiling of a branch of the internal iliac artery via a transfemoral access on the right side and surgical ligation of the obturator vein on the left side was performed. The intraoperative findings confirmed the disruption of the femoral nerve. Furthermore, additional packing retroperitoneally with four abdominal drapes and perivulvarly with one drape was done as well as the suturing of the urinary bladder and the establishment of a transcutaneous urinary diversion.

A control angiography was then performed, which showed occlusion of the external iliac artery on the left side, most likely caused by compression by the retroperitoneal packing. Reopening of the vessels was achieved by reopening the temporary wound dressings and de-packing of the pelvis. In addition, due to mesenteric ischemia, a laparotomy with a discontinuous right hemicolectomy was required.

**Fig. 1:**
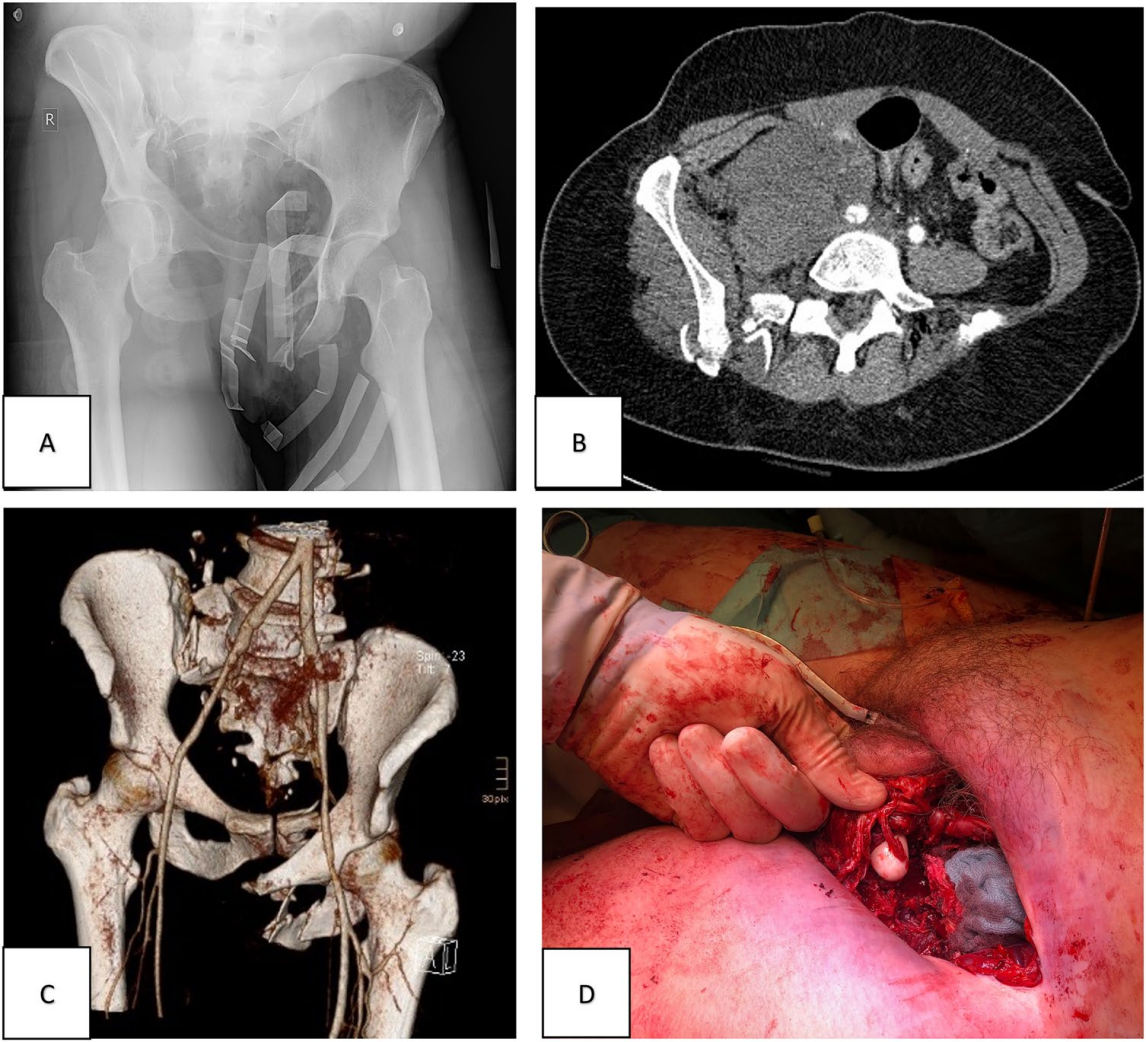
Patient 1 initial diagnostics and intraoperative findings. **A** Initial X-ray of the pelvis obtained in the emergency department under ongoing cardio-pulmonary resuscitation. **B** MS-CT scan with contrast showing a traumatic dissection of the right common iliac artery. **C** MS-3D-CT scan with contrast of the obtained pelvic injury (61-C3.2 c, h, j acc. to AO; right sacral fracture, left partial iliosacral disruption; partial traumatic hemipelvectomy), dissection of the common iliac artery and bleeding from the internal iliac artery on the right side. **D** Intraoperative findings revealing a tear of the femoral nerve

The patient died of non-occlusive mesenteric ischemia on day 4 in the intensive care unit (Fig. 1).

**Fig. 2:**
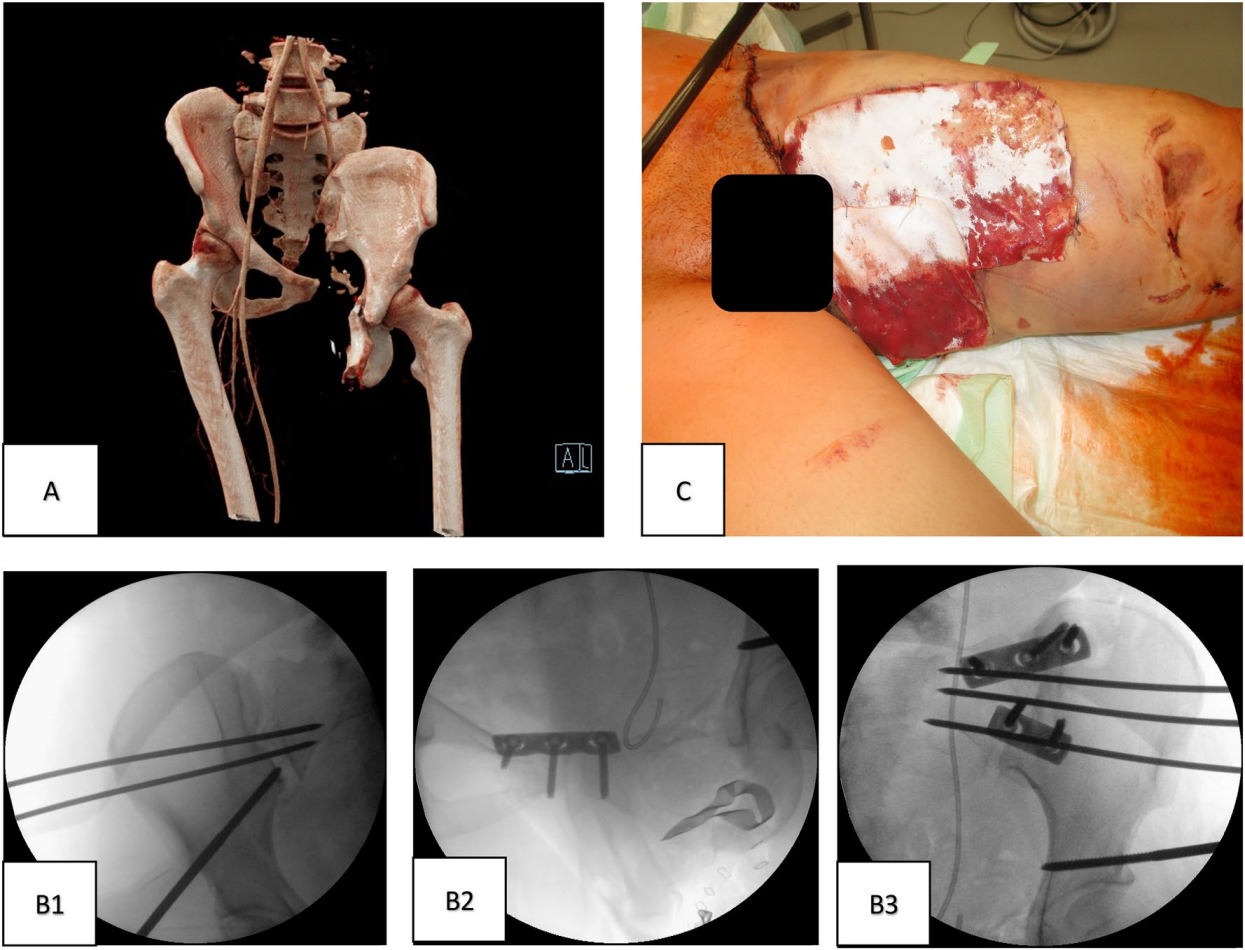
Patient 2 initial surgical strategy. **A** Initial angio 3D-CT reconstruction of the sustained injury (AO classification: 61-C3.2 a, d, j—left iliosacral disruption—right sacral fracture and 62-A1.2 acetabulum fracture on the right side; complete traumatic hemipelvectomy). **B** Closed reduction of the pelvis via supraacetabular external fixator and temporary fixation via percutaneous K-wires bilateral (**B1, B3**) and additional SI-plating on the left side (**B3**) as well as plate osteosynthesis of the symphysis pubis (**B2**). **C** Temporary wound coverage in the groin and medial thigh with Epigard^®^ (Biovision GmbH, Ilmenau Germany)

Patient 2 required resuscitation measures in the pre-hospital setting and regained adequate circulation with permissive hypotension. The right hip joint was clinically dislocated, the left side showed a high-grade soft tissue degloving injury (Morel-Lavallée lesion) and exposed iliac arterial and venous vessels. In addition, we saw a lower leg fracture of the tibia shaft (42-B3 acc. to AO) on the left side, which was immediately reduced and immobilized in an air splint. A pelvic binder was applied in the emergency room. The right hip dislocation was reduced, the vessels compressed and a polytrauma-CT performed. The patient was then transferred to the operating theatre (Fig. 2).

Inspection of the large wound on the ventral thigh revealed complete transection of the external iliac vein and artery and the left sciatic and femoral nerves. Immediate open surgical control of bleeding was accomplished and revascularization was achieved by an alloplastic graft.

Open reduction and plating was performed on the left side, while a closed reduction and stabilization with K-wires was performed on the right sacroliliac joint. The anterior pelvic ring was stabilized by plate osteosynthesis and external fixator. The peripelvic soft tissues were inspected, debrided, irrigated and temporarily covered with synthetic skin substitute (Epigard^®^, Biovision GmbH, Ilmenau Germany). In addition, a fracture of the left lower leg (42-B3 acc. to AO) was diagnosed, which was also immobilized with an external fixator.

Second looks due to abdominal trauma and ischemia were performed and a colostomy was necessary. The bladder rupture was treated by the urological surgeons. Second look for serial debridement was scheduled for the next day.

Patient 3 was admitted to the emergency department via rescue helicopter, awake, with a pelvic binder applied, analgesics administered and in a state of permissive hypotension. In the emergency department, large bore catheters were initiated and mass transfusion placed. The initial X-ray of the pelvis confirmed the suspected diagnosis of a highly unstable pelvic injury on the left side, 61-C3.1 acc. to AO.

**Fig. 3:**
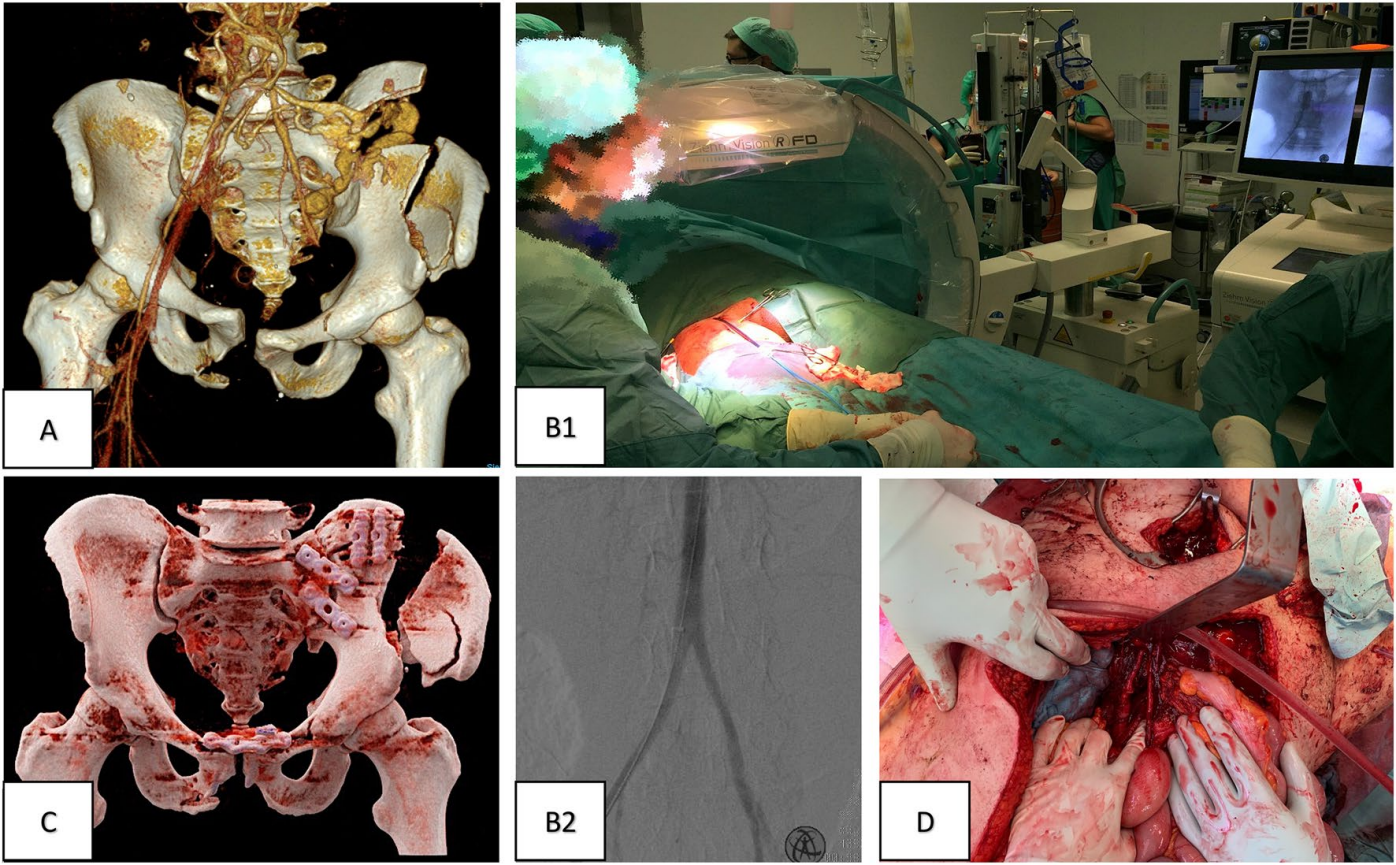
Patient 3 initial surgical strategy and control of bleeding. **A** MS-3D-CT scan with contrast of the obtained injury (AO classification: 61-C3.1 b, d, h, j—right iliosacral disruption—left multifragmentary iliac wing fracture; complete traumatic hemipelvectomy), occlusion of the external iliac artery, bleeding of the internal iliac artery. **B** Early bleeding control via clamping and packing in the resuscitation room after the application of REBOA before narcosis to prevent CPR (**B1**) and blocking on level III (**B2**). **C** Postoperative MS-3D-CT scan after vascular reconstruction, dermatofasciotomy of the left leg, and plate osteosynthesis of the pelvis. **D** Vascular reconstruction of the external iliac artery using a venous graft

As the patient initially presented hemodynamically compensated under massive transfusion, the diagnostic work-up was completed. On e- FAST examination, free fluid was detected and a polytrauma multi-slice CT scan with contrast was performed. In addition to an avulsion of the iliac vessels, severe abdominal trauma with a tear of the sigmoid colon and splenic contusion were found. After the CT diagnostics had been completed, the circulation of the patient, who was still conscious, increasingly deteriorated. The patient was immediately transferred to the OR (Fig. 3).

Due to the in extremis hemorrhagic circulatory situation, an infrarenal REBOA was placed in the OR. After blocking the infrarenal aorta (zone 3; infrarenal), a median laparotomy with retroperitoneal packing of the pelvis and reconstruction of the external iliac artery by iliacofemoral bypass was performed. The rupture of the colon was initially repaired by direct suture and a colostomy was planned in the further course. The abdomen was temporarily closed via vacuum dressing in anticipation of second look surgery and damage control procedure. The posterior and anterior pelvic rings were stabilized by plating through the same laparotomy approach.

Shortly after the patient was transferred to the ICU, a massive swollen left limb with high intramuscular pressures was noted in the compartments of the upper and lower leg. Despite massive cryotherapy, the left lower limb developed reperfusion edema with compartment syndrome due to prolonged ischemia time. The patient was immediately taken back to the operating theatre and a fasciotomy was performed for compartment decompression of the upper and lower thigh.

Patients 2 and 3 endured warm ischemia of less than 6 h. Therefore, limb salvage was attempted with vessel reconstruction in patient 2 by prosthetic replacement and in patient 3 by a venous iliofemoral bypass.

### Post-acute management

Frequent second looks (median 9; IQR 5.5; range 5–16 procedures) and wound debridement were required to control infections (both patients acquired deep wound infection). All patients were admitted to the ICU for observation and further cardio-pulmonary stabilization. Patient 1 developed severe non-occlusive mesenteric ischemia with necrotic small and large bowl three days after surgery. Therefore, palliative care because of no feasible treatment option was initiated.

Patient 2 and patient 3 were scheduled for percutaneous sacroiliac screw fixation on day 7 (2) and day 38 (3) for definitive fixation of the posterior pelvic ring.

**Fig. 4:**
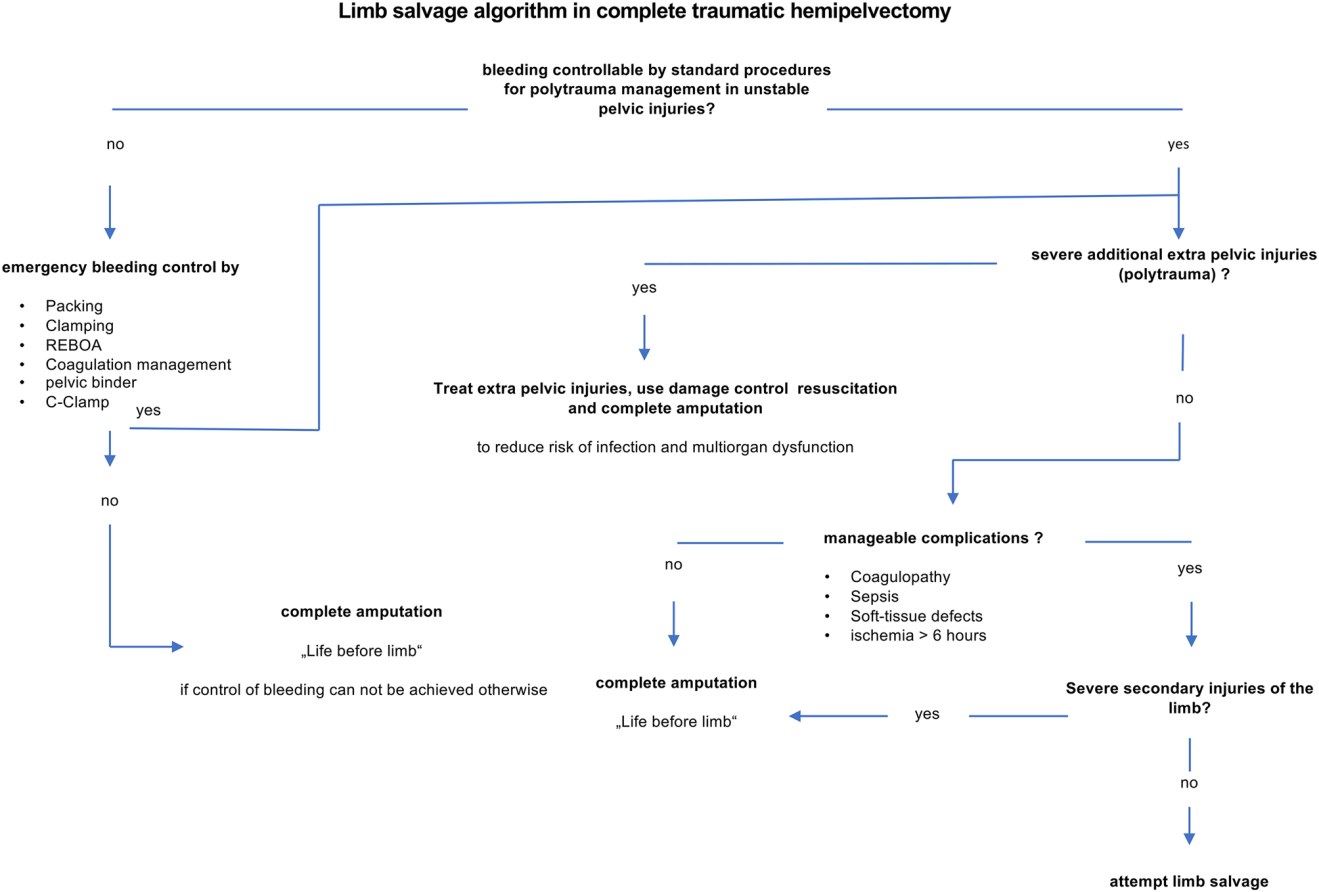
Proposal of a treatment algorithm considering limb salvage in traumatic hemipelvectomy

Limb salvage of patients 2 and 3 could be achieved. Based on our serial experience, we propose the following treatment algorithm for clinical decision-making, particularly with regard to whether limb salvage can be attempted or primary amputation is required (Fig. 4).

**Fig. 5:**
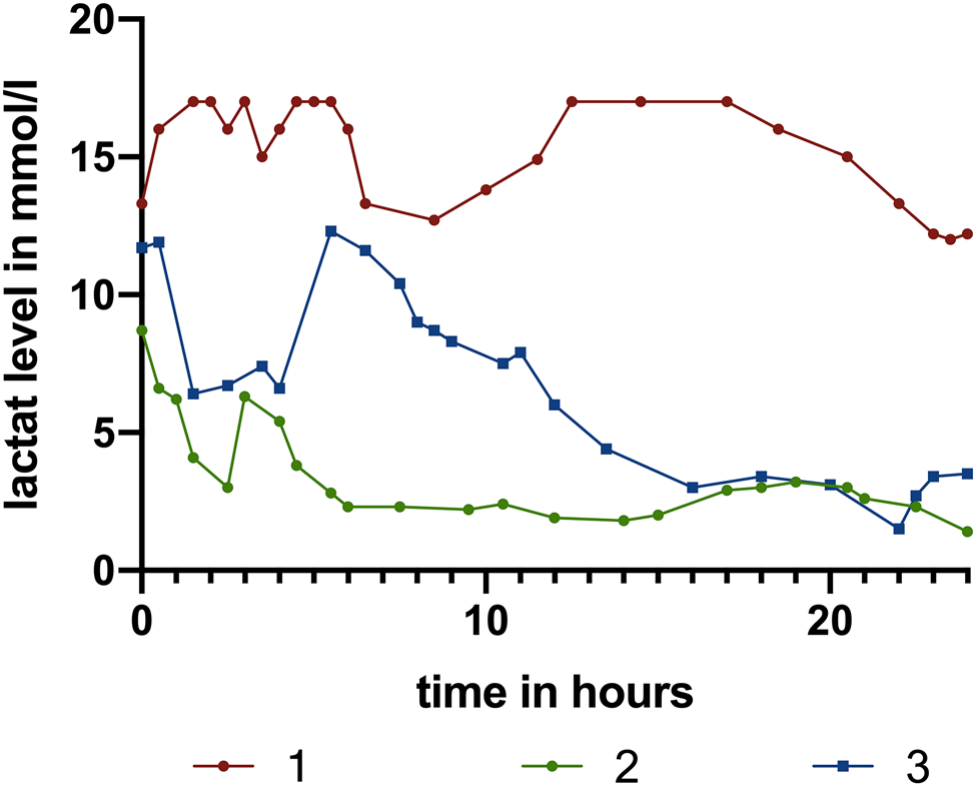
Measurements of lactate levels in mmol/l during the first 24 h following trauma. Red/green and blue: patients 1, 2 and 3. Patient 1 died due to mesenteric ischemia. The persisting high lactate level with absent lactate clearance is indicative for the outcome [37, 38]

Laboratory parameters were obtained regularly, post-traumatic temporal profile of lactate levels is shown (Fig. 5).

#### Mid-term outcome

**Fig. 6:**
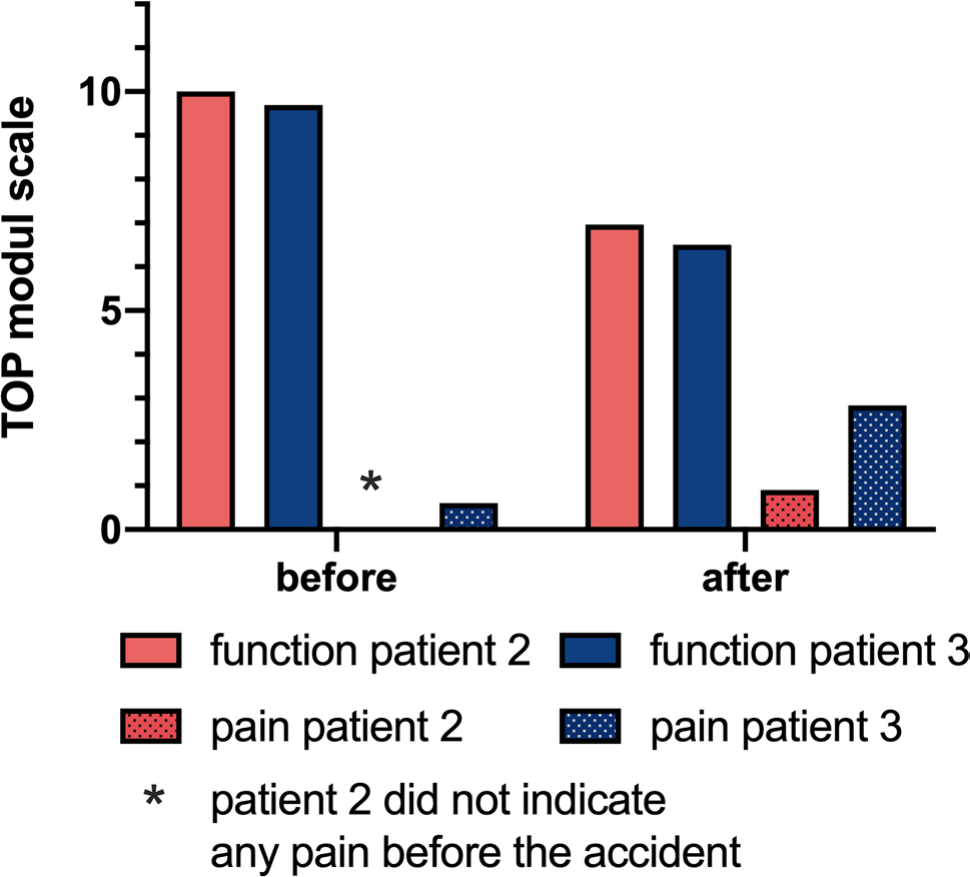
TOP-module function and pain. The mean values are shown for the first 14 items of the TOP-module; on a scale of 0 (no pain) up to 10 (severe pain)

Patients 2 and 3 were followed up approximately one year after the accident and the following charts have been obtained. Answers to the SF -36 chart and the TOP-Modul, that were answered with “to some extent/partially” and regarding pain with “moderately” were defined as abnormalities (Tables 4 and 5; Fig. 6).

**Table 4:**
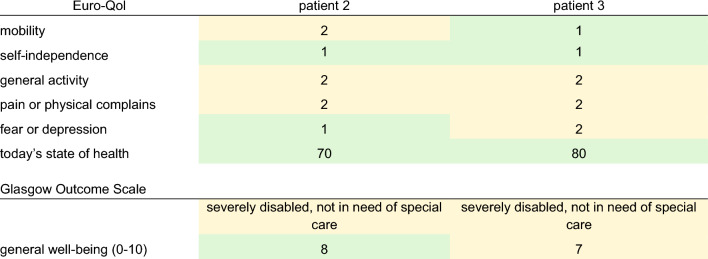
Euro-Qol-Index

First, five items on a scale of 1—no complaints; 2—some complaints; 3—severe disabilities/pain; today’s state of health on a scale of 0–100 (0—worst; 100—best). Glasgow outcome scale. General well-being on a scale of 0–10 (0—worst; 10—best)

**Table 5:**
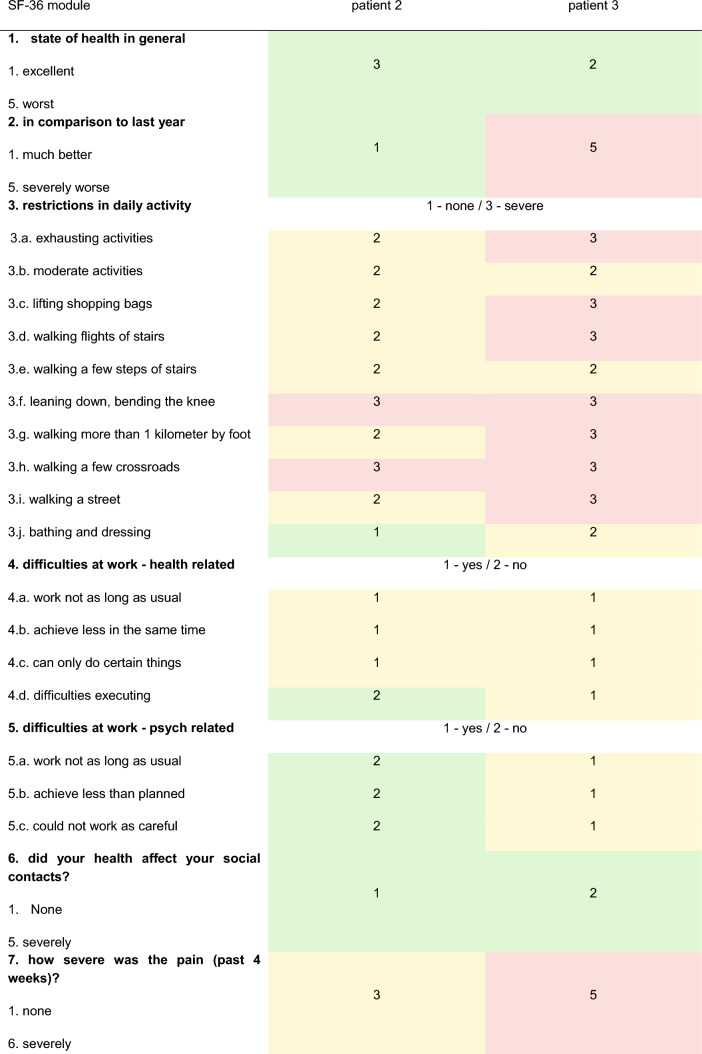

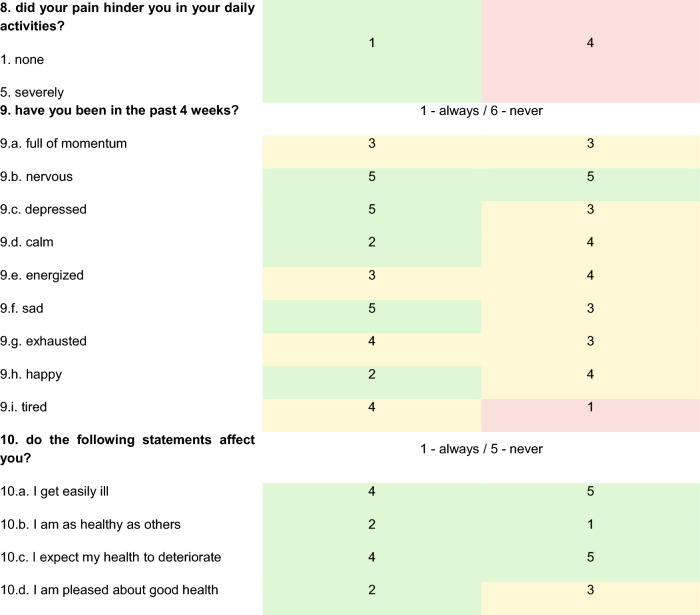
SF-36 module

**Fig. 7:**
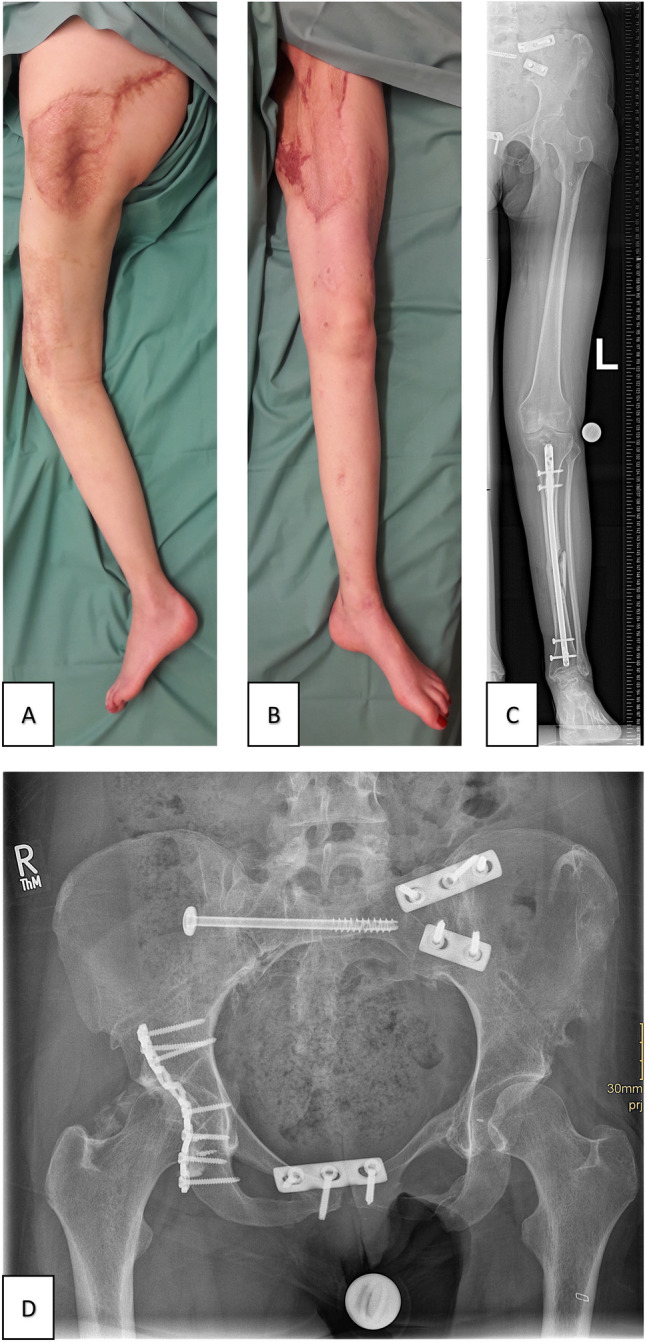
Patient 2 follow-up 18 months after the accident. **A** Clinical photographs of salvaged limb **B** Clinical photographs of salvaged limb, uneventful healing of the mesh graft. **C** Radiograph of the salvaged limb. **D** Radiograph of the pelvis

In patient 2, limb salvage could be attained. After several revisions and the transplantation of mesh grafts, the leg showed uneventful healing with stable soft tissues (see Fig. 7). Unfortunately, the leg did not regain its sensomotor function. The pain was gradually managed, the patient does not require regular pain medication at the time of follow-up and moves around in a wheelchair and on crutches; living self-independently. During manual therapy, the patient regained 20° of active hip flexion in the left leg. Now a computer-assisted motor brace, which belongs to the exoskeleton support systems, is being fitted to the patient, which could enable her to walk without crutches. The additional tibia shaft fracture on the ipsilateral side showed partial bony consolidation.

The patient uses bio-feedback to train pelvic muscles and has already achieved a significant increase in sphincter tone. Three months after follow-up, a re-anastomosis of the colostomy was performed and the patient regained complete fecal and urinary continence. Sensation in the genital area recovered and no sexual dysfunction is reported.

The patient remained psychologically stable and showed no signs of depression or post-traumatic stress disorder. Reintegration into the workplace was already initiated. The patient reported a strong social network and family support. Asking the patient whether the leg should have been salvaged or amputated in the first place, she gratefully and firmly voted for limb salvage despite immense morbidity.

In patient 3, limb salvage was also achieved. After revascularization decompressive fasciotomy was required due to the development of reperfusion-induced compartment syndrome with subsequent uneventful skin grafting (mesh graft) for wound closure. Deep vein thrombosis was treated without causing post-thrombotic problems.

The leg itself is also non-functional, with limited sensation from the waist to the knee and absent from the knee downwards. The patient moves around in an electric wheelchair and on crutches, also using a leg stabilization cuff and lives independently with regular domestic help.

Psychologically, the patient shows signs of depression, anxiety and post-traumatic stress disorder in the Top-Module and IES-R score. Therefore, the patient is in regular psychological therapy and reintegration at work was already initiated. When asked whether the leg should have been saved or amputated, the patient replied that at times he had intermittent thoughts of amputation to achieve better mobility, but finally he never doubted the decision of limb salvage as his physical integrity was preserved.

**Fig. 8:**
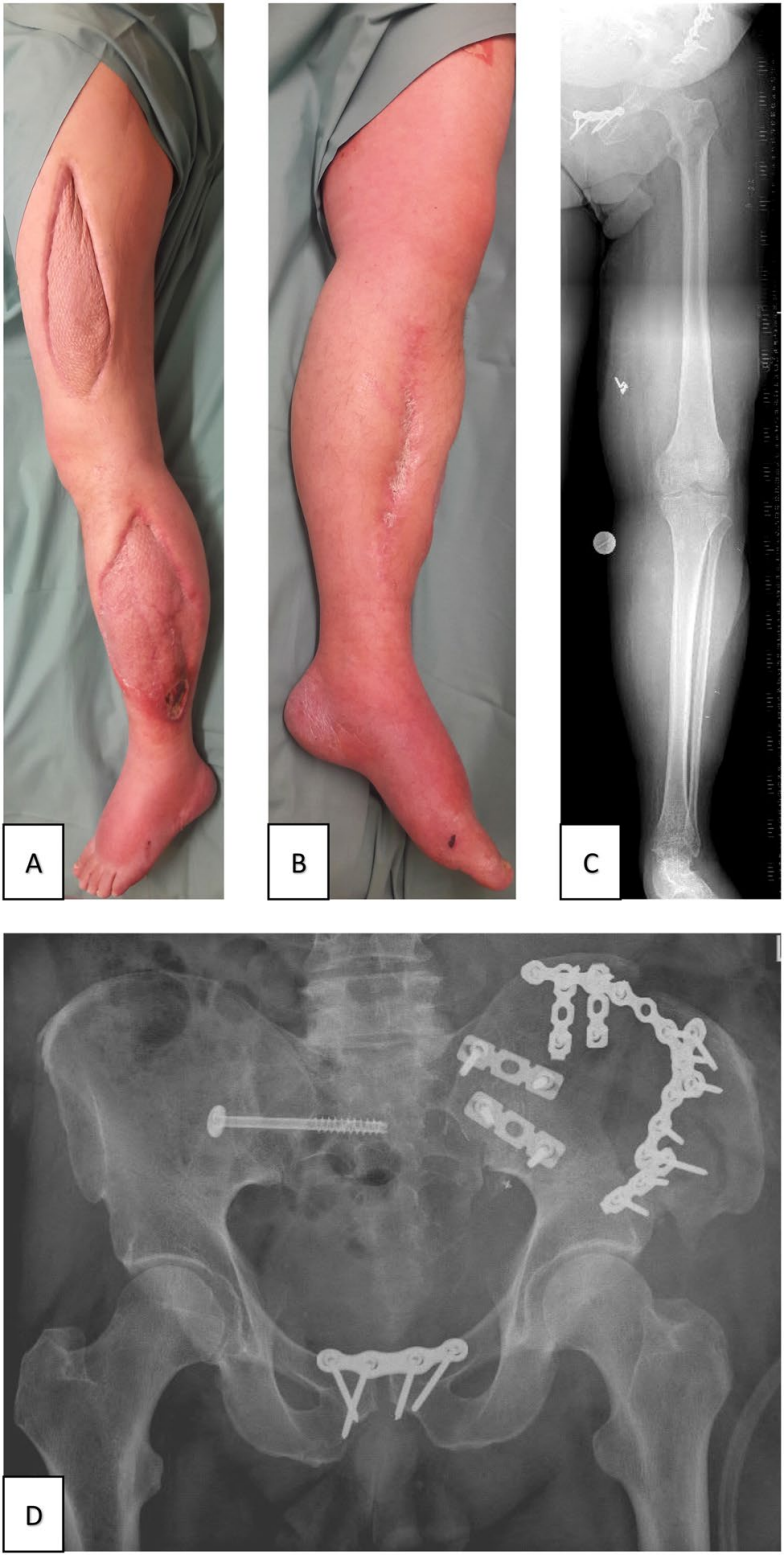
Patient 3 follow-up 14 months after the accident. **A**, **B**, **C** Clinical images and radiograph of the salvaged limb with a split-skin graft of fasciotomy. **D** Radiograph of the pelvis 4 months after the accident

At the time of follow-up, re-anastomosis of the colostomy had been achieved, urinary and fecal continence were present. Sensation and sexual function were regained in the genital area with only occasional erectile dysfunction (Fig. 8).

## Discussion

Traumatic hemipelvectomy is a rare and devastating injury with approximately 100 cases in the last decade reported worldwide. THP is difficult to treat successfully; scientifically substantiated therapeutic protocols are missing. The discussion about limb salvage, primary or early secondary amputation has been ongoing for decades [4, 6, 7, 11–13, 19, 20]. Reconstruction with limb salvage has been incidentally reported, but remained the exception [23]. Consequently, randomized multicenter studies are lacking and a universally accepted, uniform definition has not yet been established. We present a case series of three patients who sustained THP and were treated with a survival rate of 66% and successful limb salvage.

Searching for “traumatic hemipelvectomy” in the **PubMed**^**®**^ database, 47 of 92 articles were published at least 20 years ago. Improved prehospital care with invasive measures, including tranexamic acid administration, damage control resuscitation with permissive hypotension, shorter transport times [10, 14–18] and, in addition, the application of pelvic binder placement, developed in the early 2000s for prehospital stabilization of the pelvic ring [24], could lead to a higher number of patients reaching the hospital alive. Previous case reports have described different treatment options. In this case series, we share our experience in the management of this complex type of injury, report surgical as well as functional outcome results, summarize the scopes of treatment and complication profile for traumatic hemipelvectomy, and propose a treatment algorithm to facilitate decision-making for limb salvage or amputation.

### I: Bleeding control and mass transfusion protocol

The prerequisite for survival of patients with traumatic hemipelvectomy is a fast, efficient and well-functioning rescue chain. First and foremost, all patients in our case series were admitted with severe bleeding at the scene leading to massive hemorrhagic shock, which remains the leading clinical problem. Therefore, prompt emergency hemostasis and effective treatment of hemorrhagic shock in the first hours remain the major therapeutic challenge to ensure patient survival. Efficient control of hemorrhage in traumatic hemipelvectomy is challenging as blood loss may be multifactorial, e.g., arteriovenous rupture of the common iliac vessel, transection of branches of the internal iliac artery and pelvic organs, and bleeding from pelvic or sacral fractures.

Cross-clamping of the proximal aorta, iliac vessels or pre-peritoneal pelvic packing as a traditional approach for bleeding control during resuscitation are the most significant and widely established methods for acute hemostasis and temporary hemodynamic stabilization of severely injured trauma patients [39]. Some authors observed better control of bleeding in patients with complete traumatic hemipelvectomy and open soft tissue, as either clamping of the exposed vessels can be performed directly or hemostasis is achieved by thrombotic occlusion [10–13, 16].

In recent years, intra-aortic balloon occlusion has been described as a bridging method for definitive hemostatic treatment such as angioembolization or surgery [40, 41]. Some authors even proposed REBOA as a fourth pillar of damage control resuscitation [42]. Partial REBOA, a method to reduce distal ischemia and reperfusion injury by reducing the filling volume of the balloon and thereby allowing titrated low-volume aortic flow distal to the occlusion site, was developed to address and reduce ischemia-related metabolic and inflammatory risks [43]. It might provide the time needed for initial reconstruction of the damaged vessels to achieve limb salvage. On the other hand, one must also consider the limitations of the REBOA technique (especially when applied in the emergency department prior to CT diagnostics), as injuries to the aorta or pelvic vessels may be masked in the initial CT and subsequently missed (due to occlusion and prevented or severely reduced contrast medium flow), and prolonged occlusion time may cause irreversible ischemic damage to visceral or renal organs. Furthermore, Joseph et al. were able to show in their autopsy study that patients with penetrating thoracic trauma in extremis should be considered an absolute contraindication for the use of REBOA [44].

The problem that arises in severe pelvic injuries is that an intact vascular axis must be ensured to use REBOA safely. A routine CT scan with contrast and 2/3D vascular reconstructions in hemodynamically compensated patients or the performance of a pelvic radiograph, which is also available under cardio-pulmonary resuscitation, should be sufficient to identify the intact side of the pelvis in the emergency department [39].

All the above techniques were used in the management of our patients and bleeding was successfully controlled. Massive transfusion protocols were used to prevent coagulopathy [45–47]. Lactate clearance was measured constantly and showed no normalization in the non-survivor and normalized values after 24 h in the surviving patients, suggesting that lactate clearance is an important prognostic factor for survival [37, 38].

### II: Treatment of extra pelvic injuries and early surgical fixation via damage control

Previous literature describes concomitant injuries in 48% of patients with THP [9, 16]. In the present case series, all patients had concomitant injuries of varying severity, which were treated according to the damage control principles. However, the non-survivor in this case series showed extensive abdominal, thoracic and traumatic brain injuries and a significantly higher ISS.

In this context, Tiziani et al. described in their recent review of complex pelvic ring injuries that if the response to volume resuscitation is adequate and coagulopathy and bleeding are controlled, limited fixation strategies should be applied to improve outcome and reduce complication rates [48]. At our level 1 trauma center, initial surgical fixation was performed by highly experienced trauma surgeons who specialize in both pelvic surgery and polytrauma management.

### III: Management of genitourinary and anorectal injuries to avoid infection

Genitourinary injuries and anorectal lacerations are extremely common in patients with traumatic hemipelvectomies [18]. Early recognition and adequate treatment of these frequently contaminated injuries is essential to avoid wound infection [7].

If intestinal injuries, severe peripelvic soft tissue injuries or incontinence were present, a protective colostomy was performed. We believe that this approach helps to prevent difficult-to-treat infections due to fecal contamination of anorectal/perineal wounds. Re-anastomosis of the colostomy can be easily achieved following uneventful healing of these wounds. If bladder injuries were present, they were repaired in a one-stage procedure by an experienced urologist and catheterization was performed. Further injuries to the genitalia were inspected within 24–36 h after initial stabilization, with serial debridement performed as needed.

### IV: Serial debridement, intensive care therapy and definitive wound coverage

After the initial surgery, consecutive treatment in the intensive care unit is absolutely necessary. Monitoring of vital signs, renal function with urinary output and regular control of blood chemistry profile, particularly pro-inflammatory markers, are essential for early detection and treatment of multi-organ failure and sepsis. In our experience, we recommend the use of broad-spectrum intravenous antibiotic therapy and repeated serial re-debridement at 24–48-h intervals.

Another topic of controversy is whether primary wound closure or open wound treatment should be performed. Wound management remains difficult due to sometimes severe initial contamination, wound depth with large wound areas, muscle necrosis and exposed pelvic bone. Wound healing problems are reported in 75% of patients [16]. Authors advocating primary closure report (1) better mechanical stability which helps control diffuse soft tissue bleeding, (2) prevention of fluid loss, (3) protection of exposed structures, (4) creation of the prerequisite for adequate prosthetic fitting later on. Even after primary closure, a re-exploration after 48 h (2nd look) has to take place. [11, 49].

On the contrary, open wound management allows better drainage and the possibility to clean the wound frequently [3, 16]. Both we and most authors prefer open wound management [6, 10, 16] or wound-occlusive (V.A.C.) therapy [50] with secondary soft tissue reconstruction as the most effective approach to reduce the risk of secondary infection.

Modern dressings offer the advantages of primary wound closure, such as protection of exposed structures, prevention of fluid loss, conditioning of the wound and the possibility of regular debridement to reduce infectious complications [28, 51–55]. In our experience, we use primary wound closure and Epigard^©^ for initial temporary wound coverage after radical debridement. Within 24–48 h, a 2nd look with radical debridement, necrectomy and V.A.C.^©^ therapy is performed to prevent secondary wound contamination and infection and to promote wound conditioning [56].

For both temporary and definitive wound closure, early or even initial consultation with plastic surgeons is mandatory to allow planning of various soft tissue reconstructions ranging from simple secondary closure to skin grafts or local/free flap transfers. This also applies to patients requiring primary amputation. Accordingly, soft tissue defects varied widely in each of the cases we reported and wound closure was diverse [7]. The most commonly used and reported methods were myocutaneous gluteus maximus flaps, rectus abdominal flaps, latissimus dorsi flaps, composite island flaps and split-thickness grafts [4, 6, 12, 13].

### V: Provide physical and psychological aftercare for social reintegration

Survivors report traumatic hemipelvectomy as a drastic and life-changing event in their lives. When asked at follow-up, the severely limited ability to walk as well as persistent pain, incontinence and sexual dysfunction were the most decisive factors affecting quality of life [57–60].

Due to the psychological trauma, multidisciplinary care must be provided to improve psychological stability. In addition, social services and intensive rehabilitation programs must be involved to ensure social security, early reintegration into the workplace, or timely intervention with retraining measures. If possible, family and friends should be involved at a very early stage [61, 62]. The available data show that patients 2 and 3 suffered similar post-traumatic functional impairments, but assessed and managed them differently.

## Conclusion

Traumatic hemipelvectomies remain serious injuries with high morbidity and mortality despite medical advances. However, due to further improvements in prehospital, surgical, intensive care and rehabilitation care, two out of three patients survived the acute phase of trauma, demonstrating the medical progress made in recent years.

For the surviving patients, the question arises, if an attempt to salvage the limb and hemipelvis should be made if criteria are met. Certainly, the patient's life must not be endangered. To assist in the very difficult decision-making, we developed the algorithm presented here from our past experience.

The question is whether this decision to perform salvage of the extremity is also in the best interest of the patient. Weighing the impact of an amputation, i.e., hemipelvectomy, on the quality of life against an hemipelvis and a non-functional extremity (flail limb) is extremely demanding. Further studies in multicenter approach or THP-data base are needed to create more evidence.

The cases presented demonstrate the complexity of this severe trauma and the consecutive salvage of the limb and hemipelvis. Therefore, the question of limb salvage still remains an individual decision, requiring enormous experience both in the treatment of pelvic injuries as well as polytraumatized patients.
